# Topographic heterogeneity lengthens the duration of pollinator resources

**DOI:** 10.1002/ece3.6617

**Published:** 2020-08-10

**Authors:** Rachael L. Olliff‐Yang, David D. Ackerly

**Affiliations:** ^1^ Integrative Biology UC Berkeley Berkeley CA USA; ^2^ Environmental Science, Policy, and Management UC Berkeley Berkeley CA USA

**Keywords:** community, flowering period, flowering time, microclimate, phenological asynchrony, phenology, plant phenology, plant-animal interactions, pollinator resources, resource duration, topography

## Abstract

The availability of sufficient and diverse resources across time is important for maintenance of biodiversity and ecosystem functioning. In this study, we examine the potential for variation in environmental conditions across topographic gradients to extend floral resource timing. Flowering time on a landscape may vary across topography due to differences in abiotic factors, species turnover, or genotypic differences. However, the extent to which this variation in phenology affects overall flowering duration on a landscape, and the components of diversity that influence flowering duration, are unexplored. We investigate whether differences in flowering time due to topography yield an overall extension in duration of flowering resources in a northern California grassland. We recorded flowering time of pollinator resource species across four successive spring growing seasons (2015–2018) on paired north and south aspects. Flowering time differences were evaluated both at the community level and within species present on both paired aspects. The role of plasticity was examined in an experimental case study using genotypes of *Lasthenia gracilis*. We found that aspect is a strong determinant of phenology, with earlier flowering on warmer south‐facing slopes. Aspect differences resulted in complementarity in timing of flowering resources across sites, as aspects that started flowering earlier also ended earlier. Complementarity between north and south aspects served to extend the flowering time of pollinator resources by an average of 4–8 days (8%–15%), depending on the year. This extension can be attributed to both within‐species responses to aspect differences and species turnover. Flowering of *L. gracilis* genotypes was distinct across aspects, demonstrating that plasticity can drive the extension of flowering duration. Our findings indicate that heterogeneous topography can extend overall flowering time of pollinator resources, which may support pollinator biodiversity. Extension was most pronounced at the community level, which incorporates species turnover as well as plastic and genotypic differences within species.

## Graphical abstract

We examine the potential of variation in environmental conditions across topographic gradients to extend the duration of floral resources for pollinators. Aspect is a strong determinant of flowering time, and complementary flowering on paired north‐ and south‐facing slopes extended the duration of pollinator resources by an average of 4–8 days (7%–15%), depending on the year. This extension can be attributed to both intraspecific and interspecific responses to aspect differences.

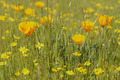

## 1 INTRODUCTION

**FIGURE 1:**
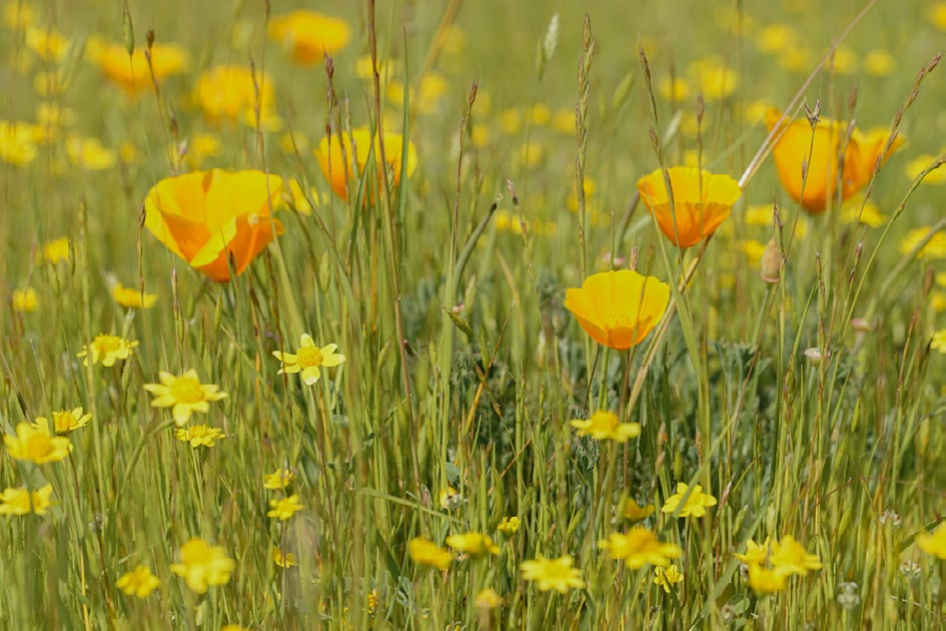
A grassland community at Pepperwood Preserve (Sonoma County, California, 38.57°N, −122.68°W). Our study site was a heterogeneous Mediterranean‐type grassland with a rich array of pollinator resource species, both native and non‐native. Pictured are the iconic California poppy (*Eschscholzia californica*, Papaveraceae) and common goldfields (*Lasthenia gracilis*, Asteraceae), among other grass and forb species. Photograph by Alexander C. Yang

Sufficient and diverse resource availability across time is important for biodiversity and ecosystem functioning. Resource availability is dependent on the phenology (seasonal life cycle timing) of both resources and interacting partners in a system. For pollinators, the presence of pollen and nectar‐rich floral resources (e.g., Figure 1) across the entire flight season is critical for maintaining diversity, population stability, and pollination function (Russo, DeBarros, Yang, Shea, & Mortensen, 2013). A reduction or change in season duration can have adverse consequences for both pollinator and plant populations (Aldridge, Inouye, Forrest, Barr, & Miller‐Rushing, 2011).

Anthropogenic climate change may shorten phenological duration (Høye, Post, Schmidt, Trøjelsgaard, & Forchhammer, 2013; Prevéy et al., 2019) posing a risk to pollination mutualisms. On the other hand, an extension of flowering time duration can support mutualisms (Hindle, Kerr, Richards, & Willis, 2015), and increase local pollinator biodiversity and pollination efficacy (Morandin & Kremen, 2013). Climate refugia (locations on the landscape where the impacts of climate change are buffered) are expected in heterogeneous landscapes, due to the presence of a variety of microclimatic conditions (Morelli et al., 2020). We predict that variations in microclimate created by heterogeneous topography might also aid plant–pollinator mutualisms by serving to extend flowering duration across space. This prediction necessitates a better understanding of the influence of topography on flowering time on the landscape.

The timing of flowering is driven strongly by abiotic cues, including temperature, moisture, and photoperiod (Rathcke & Lacey, 1985). As temperature and precipitation have shifted with climate change, so have the timing of life history events, advancing the timing of flowering and pollinator foraging seasons (Parmesan, 2006). Climate change has already led to species‐specific timing changes in plants and pollinators (CaraDonna, Iler, & Inouye, 2014). These shifts have been documented to disrupt species interactions (e.g., Schmidt et al., 2016) and can result in pollination asynchronies, especially in free‐living mutualistic partners with brief seasonal interactions (Rafferty, Caradonna, & Bronstein, 2015).

Phenological responses to abiotic conditions can also lead to timing differences across gradients on a landscape (Ward, Schulze, & Roy, 2018). Topoclimate, or small‐scale (10–100 m) variations in abiotic conditions due to differences in topography (Geiger & Aron, 2009; Oldfather et al., 2016), can be used to observe the combined effects of differences in temperature and moisture gradients on phenology. Topoclimate differences can greatly influence individual species phenological timing, and in some cases, the magnitude of variation in timing across a landscape is greater than interannual variation due to yearly weather conditions (Weiss, Murphy, Ehrlich, & Metzler, 1993; Weiss, Murphy, & White, 1988). Slope and aspect temperature and moisture differences can be ecologically significant across even moderate topography, driving vegetation patterns and ecosystem processes (Bennie, Huntley, Wiltshire, Hill, & Baxter, 2008). These effects may influence community flowering time across the landscape by affecting individual species timing and species turnover.

Topography can create short‐distance gradients in abiotic conditions comparable to those observed across larger latitudinal or elevational gradients. North‐facing slopes in the northern hemisphere are pole facing and therefore receive lower amounts of incident solar radiation (i.e., insolation). Equator‐facing slopes, or south‐facing slopes in the northern hemisphere, receive more direct solar radiation and therefore experience higher temperatures and a faster soil drydown rate (Bennie, Hill, Baxter, & Huntley, 2006). These energy load differences between north‐ and south‐facing slopes are greatest in the midlatitudes, due to planetary geometry (Holland & Steyn, 1975). The dynamics of light, temperature, and moisture differences across these two contrasting aspects allow for the examination of different abiotic environments in close proximity.

**FIGURE 2:**
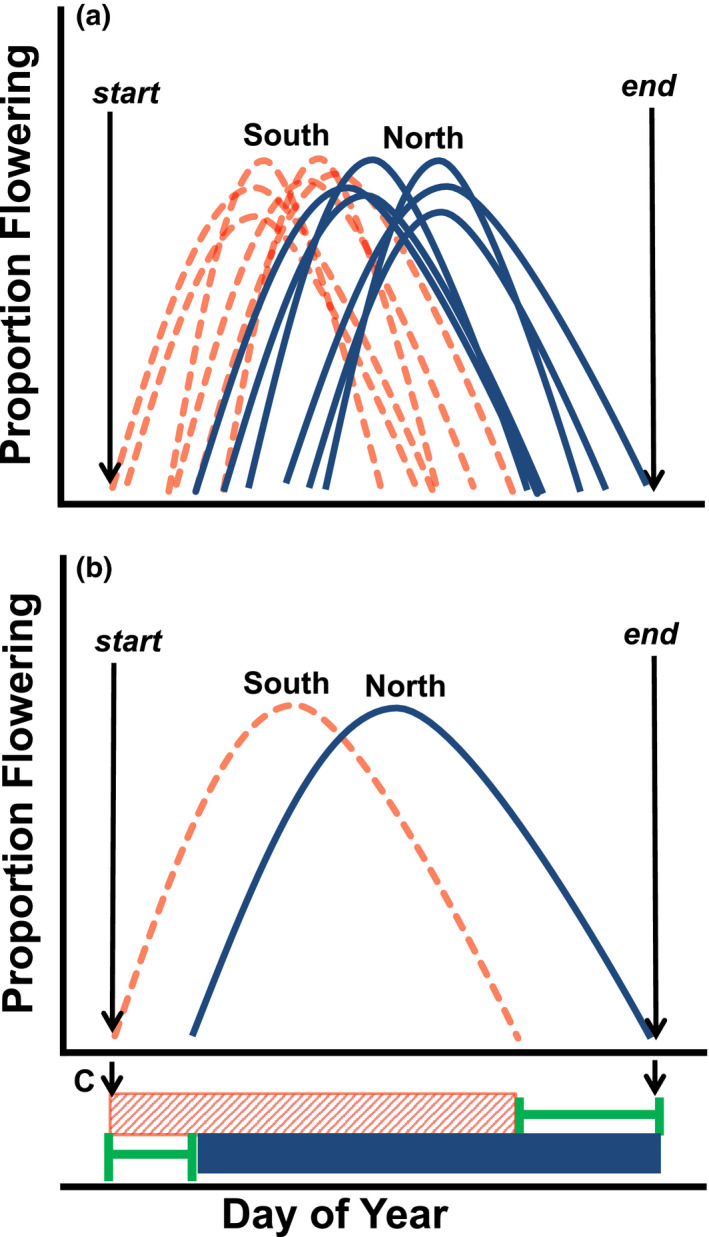
Theoretical overlap of flowering time on two adjacent opposite‐facing slopes. Proportion of flowering is (# flowers each date)/(total season flowers). Here, light red (dashed/patterned) indicates south facing, and dark blue (solid) indicates north facing. (a) Individual species flowering curves on each slope, (b) cumulative flowering proportions of all species on each slope, and (c) overall flowering time duration, and extension due to complementarity. North‐facing slopes in the northern hemisphere may have a delayed phenology due to lower amounts of incident solar radiation. The flowering time across these two adjacent areas on a landscape may yield a longer overall flowering time on the landscape than in either location individually due to complementarity (nonoverlap) in resources. This extension in flowering time duration due to complementarity is shown by the bold green brackets (c)

Varying abiotic conditions due to small‐scale heterogeneity in topography can cause patch differences in flowering time, yielding an overall extension of flowering across the landscape. For example, each aspect on a hill will have a start, middle, and end date of flowering time (Figure 2a,b). The duration of flowering on the landscape will be determined by the complementarity (or nonoverlap; e.g., Figure 2c, green brackets) between these phenological curves, from the earlier of the start dates to the later of the end dates (see Figure 2). Therefore, the duration and degree of complementarity in flowering time across topographic gradients on the landscape determines the overall flowering time. Just as herbivores can “surf” waves of green‐up across the landscape (Merkle et al., 2016), complementarity in flowering time among aspects may allow pollinators to utilize flowering resources available over time in different patches on the landscape.

Extensions in flowering time can be generated by population plasticity, genotypic heterogeneity (Smith et al., 2015), and species turnover (Timberlake, Vaughan, & Memmott, 2019; reviewed in: Olliff‐Yang, Gardali, & Ackerly, 2020). By measuring both community‐level and species‐level components of flowering differences across topographic gradients, we can examine the components of diversity that contribute to observed patterns of flowering time. Both plasticity and genotypic variation can contribute to intraspecific differences in phenology across topoclimates (Anderson, Inouye, McKinney, Colautti, & Mitchell‐Olds, 2012; Phillimore, Stålhandske, Smithers, & Bernard, 2012). Species turnover will yield additional changes in the timing of flowering resources (Wright, Vanderbilt, Inouye, Bertelsen, & Crimmins, 2015). The effects of these components may be antagonistic across the landscape, yielding similar timings, or synergistic, yielding an extension in timing when complementary flowering patches are combined together. The combined effect of both community‐level and species‐level components will influence the overall flowering time at the community level.

It is valuable to examine the different influences of topographic heterogeneity within and among species, as the components will matter for plant–pollinator mutualisms. Insect species can specialize on specific plant taxa or on plant species with similar traits across clades (Willmer, 2011). Specialist pollinator species depend on the flowering time of only the particular taxa or morphological type of plants they visit (Willmer, 2011). If topographic gradients result in an extension of flowering duration within species, then specialist pollinators will benefit from complementarity (Olliff‐Yang et al., 2020). In contrast, if an extension in flowering time is driven by species turnover, then the benefits will depend on the spectrum of pollinators utilizing the respective plant species. The scale of floral resource availability on the landscape matters, as small pollinators may only move short distances while foraging (≤100 m), while larger pollinators can forage over longer distances (e.g., bumblebees up to 1.5 km) (Osborne et al., 2008; Zurbuchen et al., 2010). Patches of floral resources on the landscape must be present within foraging range to benefit pollinators.

Mediterranean‐type climates are characterized by cool wet winters and hot dry summers (Köppen, 1923). In the grasslands of California, these thermal dynamics and timing of precipitation yield a lush green landscape in the winter, a colorful flowering period from March to June, and a senescent period in the summer as the soil dries out (Dallman, 1998). These timing dynamics play out in different ways across the landscape depending on the temperature, moisture, and light available at each point. Species‐specific differences in flowering time also contribute to variation across the landscape, leading to complementarity in utilization of soil nutrient and moisture resources across the season (Gross, Suding, Lavorel, & Roumet, 2007; Wolkovich & Cleland, 2011).

In this study, we compare the flowering time of a grassland community (Figure 1) across paired north‐ and south‐facing slopes, and examine the components of diversity involved in the flowering responses to topoclimatic conditions. We investigate how heterogeneous topography influences the duration of flowering time across the landscape, and decompose the components that may lead to an extension in timing within and among the grassland species. Specifically, we address the following: (a) How does microsite variation due to aspect impact the flowering time of pollinator resources? (b) Do differences in flowering time due to topography yield an overall extension of flowering resources on the landscape? (c) What is the contribution of differences within versus. among species to observed patterns at the community level? (d) When controlling for genotypic differences, does plasticity contribute to intraspecific differences across aspect? We explore these questions to assess the importance of topography on community‐level pollinator resource timing, and to evaluate the potential for topographic heterogeneity to mitigate shifts in phenology with climate change. Adaptive capacity, the ability to respond to climate changes via evolutionary, plasticity, or dispersal events, will increase a species chance of survival into the future (Beever et al., 2016). Extended phenological timing has been proposed as a possible way to buffer some impacts of shifts in phenology with climate change, yielding adaptive capacity (Olliff‐Yang et al., 2020). If topographic heterogeneity lengthens flowering time duration on a landscape, it may serve to support species interactions in responding to climate changes.

## 2 MATERIALS AND METHODS

### 2.1 Study system

This study was conducted in the grasslands of Pepperwood Preserve (Figure 1; Sonoma Co., California, 38.57°N, −122.68°W). Four sites were chosen based on presence of paired north (pole‐facing) and south (equator‐facing) aspects within 100 m of each other (35–80 m), and with pollinator resource species present (M. Halbur, pers. comm.). The sites were located in grasslands within 2 km of each other. On each aspect, three 1 m^2^ quadrats were placed randomly, and spaced 3 m apart, yielding a total of 24 plots. In 2016 and 2017, an additional site was monitored (*n* = 30 plots in these years).

### 2.2 Abiotic measurements

Temperature and moisture were recorded at the plots to quantify microsite differences between aspects. Temperature was recorded at each aspect with an iButton (Thermochron, *N* = 8) placed 10 cm below the soil surface and set to record every hour. Temperature measurements were taken from March through June in all 4 years to compare aspect temperature differences during the flowering season. Additionally, temperature measurements were collected throughout the year in 2016 to capture full growing degree‐day accumulation curves. Soil moisture was measured manually in 2016 (on 26 Apr, 26 May, 21 Jun) and 2017 (on 10 Mar, 6 Jun) at all plots with a Hydrosense II soil moisture probe (Hydrosense II (Campbell Scientific, Inc.), 2019). Temperature measurements were taken simultaneously, and moisture measurements were taken within 10–30 min of each other at each site on each measurement date. For site temperature comparisons, any missing temperature data (e.g., an ibutton failure in 2017, animal disturbance of an ibutton in 2018) were extrapolated by taking site averages from other years and adjusted based on air temperature differences. Cumulative temperature differences were assessed by visually comparing growing degree‐day accumulation (using base temperature of 5°C) and tested using a binomial sign test. Differences in maximum temperatures, minimum temperatures, and soil moisture on north and south aspects were assessed using analysis of variance (ANOVA) models, with site, measurement date, and year as fixed effects. Moisture data were log transformed to meet assumption of residual normality. Significance of aspect was assessed via model comparison with simplified models (with aspect removed).

### 2.3 Phenology measurements

In each plot, we recorded flowering throughout the spring growing season (March–June) in 2015, 2016, 2017, and 2018. Flowering phenology was observed for all pollinator resource species in the plots, including native and non‐native species, annuals, and perennials. Species status as a pollinator resource was identified by direct observations of animal visitation during the study, together with outside sources, including information provided by the Xerces society (Mader, Shepherd, Vaughan, Hoffman Black, & LeBuhn, 2011). Richness of pollinator resource species in each plot varied from 1 to 22 species over the entire season. Inflorescences in flower for each species in each plot were counted weekly to determine start, middle, and end flowering, as well as the length of the flowering season. For species with inflorescences that had more than one phenology stage present, an inflorescence was counted as flowering when at least 50% of it was in flower. Not all species were present in all sites, or on both slopes.

Community flowering dates for pollinator resource species were calculated based on cumulative plot flowering over the season, as follows: start date as the date when 5% of the cumulative number of flowers in a plot (summed over the season) had been reached, midflowering date as the date when 50% flowering was reached, and end date as the date when 95% of flowering had been reached. Flowering duration was defined as the total number of days between start and end dates (when 5% and 95% flowering had been reached, respectively) for each plot.

### 2.4 Analyses

#### 2.4.1 Q1: microsite variation in resource timing

The relationship of midflowering to average temperatures during the flowering season (March–May) was tested using linear regression models, examining both within‐year and between‐year trends. Sites (and years in the combined model) were included as fixed factors to account for plot pairing. Flowering dates were then compared across north‐ and south‐facing aspects, with aspect, year and site as fixed factors. As it was not monitored in 2015 or 2018, the fifth site (TT, three tree hill) was not included in these interannual ANOVA comparisons, to maintain a balanced design. Models were then tested against simplified models (with aspect removed) to determine whether aspect was significant in determining flowering date and compared using AICc information criterion metrics. As the effect of aspect may differ depending on the year and site, interactions with aspect were also tested by comparing full ANOVA models against models with interactions removed.

#### 2.4.2 Q2: phenological extension

Extension in flowering time was calculated by comparing the duration (in number of days) of flowering time on both aspects combined at a site for each year, versus the duration on the longer of the two slopes (north or south). This is a conservative calculation of flowering time extension, because it is based on extending the longer flowering slope, and duration start and end dates were defined as the date of 5% and 95% flowering, respectively (see above). The percent of flowering resource extension was calculated as:

$$\text{Extension}=\frac{\text{Combined}-\text{Longer}}{\text{Longer}}*100$$

A binomial sign test was performed to examine the influence of aspect on complementarity, testing whether aspects with earlier flowering start dates also end earlier more often than expected by chance.

#### 2.4.3 Q3: diversity components of extension

Absolute turnover at a site was calculated as the total number of species present on only one aspect (i.e., Turnover = [# unique species on N aspect] + [# unique species on S aspect]). This was calculated for each site/year combination. To examine the amount of extension explained by community turnover, absolute turnover was compared with observed flowering time extension using simple linear regression.

Extension in community flowering time was then examined with species turnover removed, to decompose the influences of flowering time differences between aspects. To do this, the community‐level analyses were restricted to only include species present on both aspects at a site in a given year. The community flowering dates for pollinator resource species were then recalculated based on cumulative plot flowering over the season. In this analysis, any difference in flowering time observed between aspects is due to differences in within‐species responses (plasticity or genetic variation), and not attributed to species turnover at the site.

Aspect influence on flowering time was also examined at finer scales. To assess the extent to which the flowering time of individual species was affected by aspect, the difference in flowering dates was calculated for each species present on both slopes at a site. Species flowering dates were defined as above: start date as the date when 5% of the cumulative number of flowers in a plot (summed over the season) had been reached, midflowering date as the date when 50% flowering was reached, and end date as the date when 95% of flowering had been reached for each species. When flowering was only observed for a species on one survey date, the duration of flowering was calculated as 1 day (although it is likely that flowering occurred for 22 days or longer depending on the species). There were instances of gaps between flowering time on north and south aspects for individual species, and these were removed when calculating site flowering duration. The fifth site (monitored in 2016 and 2017) was included in the species‐level comparison of flowering date differences and season extension, as it added 3 new species and additional observations of other species. However, this additional site was not used in any ANOVA model comparisons.

#### 2.4.4 Q4: population plasticity contributions to differences across aspect

Sites were chosen with paired aspects in close proximity (<100 m), and therefore, the genetic differentiation between north and south slopes was expected to be minimal. However, to explicitly examine the role of plasticity in aspect effects, experimental plots of goldfields (*Lasthenia gracilis* (DC.) Greene) were set up just outside of phenology plots in 2017 and 2018, with 3 subplots per aspect at each site (*n* = 24). These 30 × 30 cm subplots were planted with 30 seeds each, collected from two grassland locations on Pepperwood Preserve from 10 maternal lines (3 seeds per line per plot). Seeds were planted in the fall and marked with toothpicks to differentiate them from any other *Lasthenia* individuals occurring at the site. Flowering time was recorded for these subplots in the same way as the study phenology plots—with all open inflorescences from all individuals counted each week from March through June. Counts were conducted only on planted and marked *Lasthenia* individuals within each plot. As plot flowering was composed of individuals from the same genetic lines, any differences in timing between north and south aspect plots would therefore reveal population mean plasticity across aspects. Flowering time of *L. gracilis* within each site (due to presence on both aspects) was assessed using ANOVAs. Flowering time and extension metrics were quantified and analyzed using the same method as the community‐level and species‐level analyses. All analyses were performed in version 3.6.2 of R, using tidyverse packages in RStudio (R Core Team, 2019; RStudio Team, 2020; Wickham et al., 2019).

## 3 RESULTS

### 3.1 Q1: microsite variation in resource timing

**FIGURE 3:**
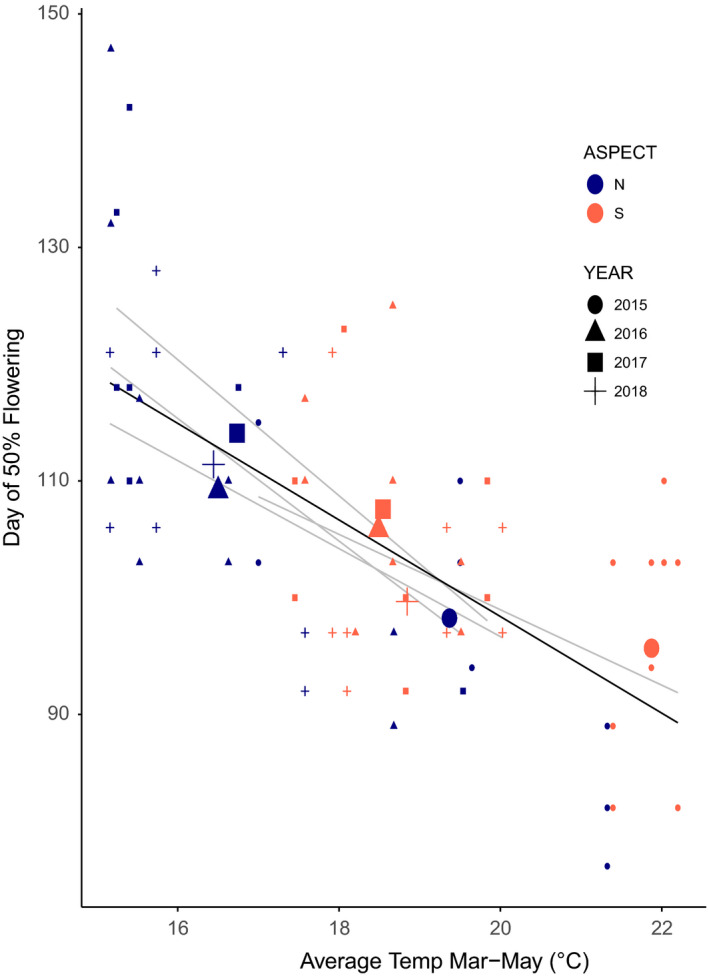
Date of midflowering by average temperature during the flowering season. Colors indicate the plot aspect: north (dark blue) and south (light red). Symbols indicate years: 2015 (circles), 2016 (triangles), 2017 (squares), and 2018 (crosses). Gray lines of fit are from simple linear regression of midflowering date by average March–May temperature in each year. Black line shows the overall relationship between the midflowering date of pollinator resources to average plot temperature during the flowering season across years (slope = −3.4, *R* ^2^ = 0.55, *p* < .001, [from full linear model with year and site included as covariates], marginal *R* ^2^ of temperature: 0.41). Larger points show overall means of each aspect(color)/year(shape) combination (e.g., blue triangle is the overall mean of north aspects in 2016) for visual comparison of overall aspect differences each year

Aspect was significant in determining both maximum and minimum temperatures (*p* < .001). Soil temperatures on north‐facing slopes were on average 3.06°C cooler than south‐facing slopes. This led to warmer south aspects overall (Figure 3—compare overall aspect/year points), and a faster accumulation of growing degree‐days on south‐facing slopes (Figure S1; S aspects accumulated more growing degree‐days March–June than N aspects [in 17/18 cases; binomial test, *p* < .001]). Paired aspects had significantly different soil moisture content (*p* < .001), with north aspects more moist (approximately 1.7% VWC higher on average than south‐facing slopes on measurement dates). However, one site (BH) tended toward lower volumetric water content on the north‐facing slope, likely due to thinner soils on this aspect.

**FIGURE 4:**
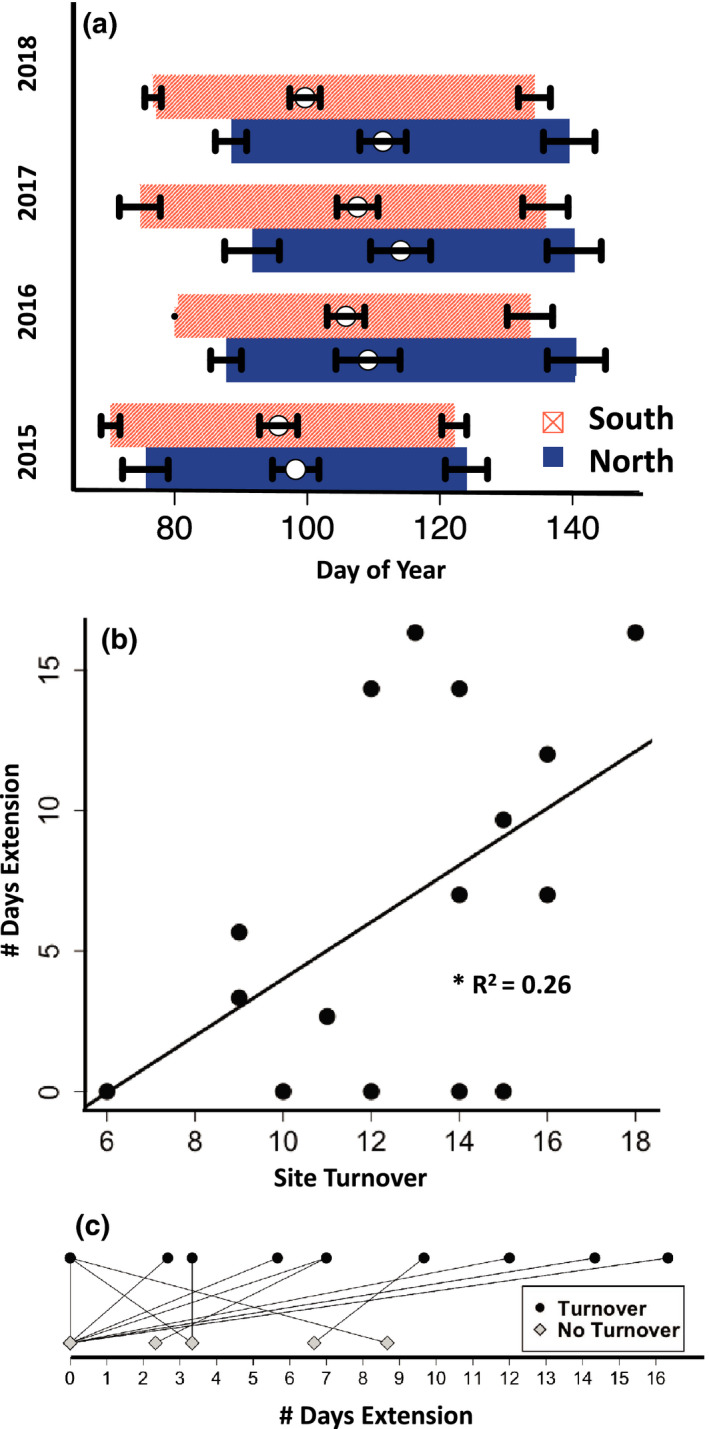
Topographic influence on flowering duration. (a) Start (date of 5% flowering, left end), midflowering (date of 50% flowering, white middle points), and end (date of 95% flowering, right end) of flowering time of pollinator resources in plots on south (light red patterned) and north (dark blue solid) facing slopes in 2015–2018. Day of year (DOY): DOY 80 = 21 March, DOY 100 = 10 April, and DOY 140 = 20 May [+1 DOY for 2016 leap year]. Black bars show standard error of the mean. (b) Number of days extension of pollinator resource flowering time by absolute site turnover. Turnover was calculated as the total number of species found on only one of the two aspects at a site in a given year (Turnover = # unique species on N aspect + # unique species on S aspect). Points show the number of days extension of pollinator resource timing at a site in the full community (all species, with turnover). (c) Black points show the number of days extension of pollinator resource timing at a site in the full community (all species, with turnover), and gray‐filled diamonds show community flowering time extension calculated without species turnover (restricted to only pollinator resource species present on both aspects at a site). Line segments connect site/year pairs with versus without turnover calculations. The number of days of extension was reduced in 10/16 sites, 4/16 remained the same, and 2/16 increased when turnover was removed from extension calculations

Aspect was a strong determinant of phenology for the start, mid, and end of flowering (Table 1). Warmer plots (due to warmer slopes and/or years) resulted in earlier timing of pollinator resources in all years (Figure 3; slope = −3.4, *R* ^2^ = 0.55, *p* < .001). Flowering time differed between aspects, with earlier timing on south‐facing slopes (Figure 4a). These phenological responses to landscape position resulted in differences in flowering time leading to complementarity across slopes (Figure 4a), as aspects that started flowering earlier also ended earlier more often than expected by chance (binomial test, *p* = .021). Year was also significant in determining start, mid, and end dates (all dates *p* < .001), likely due to differences in temperature and precipitation each year (Figure S2). However, the interaction between year and aspect was not significant, so aspect influenced timing similarly every year. The effect of aspect on flowering time was dependant on the site (Site:Aspect interaction *p* < .001), and this site effect was consistent across years (Year:Site:Aspect interaction NS, Table S2).

**TABLE 1:** Testing aspect influence on flowering time

Phenology Measure	Model	*df*	AICc	*R* ^2^m	*R* ^2^c	*F* Ratio	*p* value
Start	null (Y + S)	8	714	0.335	0.290	42.7	<.001***
	Y + S + **Aspect**	9	679	0.552	0.516		
Mid	null (Y + S)	8	729.7	0.445	0.408	9.1	.003**
	Y + S + **Aspect**	9	722.6	0.497	0.457		
End	null (Y + S)	8	750.6	0.303	0.256	4.0	.047*
	Y + S + **Aspect**	9	748.8	0.333	0.280		

Note

Testing the inclusion of aspect as a fixed effect in the models, against the simplified model with aspect removed. Models include dates (Start [date of 1st 5% flowering], Midflowering [date of 50% flowering], and End [date of last 5% flowering]) as dependant variables, with year (Y) and site (S) as fixed effects. *F* ratios, *p* value, and and bold font, asterisks (*p* < .001***, *p* < .01**, *p* < .05*) indicate significance of including aspect in the model, as determined by testing the full model against the null model with aspect removed.

### 3.2 Q2: phenological extension

Where complementarity in community flowering time existed between slopes, it served to extend overall flowering time of pollinator resources by approximately 4–8 days (8%–15%), depending on the year [mean 6.8 days, or 12.1%] (Table 2A). Having both aspects present at a site increased the duration of flowering time in most sites (Table S1). The coolest and wettest year (2017) yielded the longest overall combined community flowering duration across aspects, mainly due to a lengthened flowering duration on the south aspects (Figure 4a, Table 2A). However, the interaction between year and aspect was not significant.

**TABLE 2:** Overall community duration of season and extension metrics

(A) Community pollinator resources						
Year	Mean south	Mean north	Mean combined	Mean days extension	Range of days extension	Mean extension
2015	51.8	48.4	58.4	4.42	0–14.3	7.9%
2016	53.6	52.8	64.6	7.25	0–16.3	12.7%
2017	61.2	48.6	70.1	8.33	0–14.3	15.2%
2018	57.5	51	65.3	7.17	0–16.3	12.6%

(B) Community pollinator resources, no turnover						
Year	Mean south	Mean north	Mean combined	Mean days extension	Range of days extension	Mean extension
2015	40.58	45.67	50	3.83	0–8.7	8.63%
2016	51.67	50.83	54.58	0	0	0.00%
2017	40.83	42.5	53.08	0.58	0–2.3	1.00%
2018	49.58	48.33	56.33	1.67	0–6.7	2.72%

(C) Extension difference		
Year	Site	Extension Difference (days)
2015	BH	0.00
2015	DP	−8.67
2015	FR	−3.33
2015	TP	12.67
2016	BH	0.00
2016	DP	9.00
2016	FR	2.00
2016	TP	16.33
2017	BH	0.00
2017	DP	4.67
2017	FR	14.33
2017	TP	12.00
2018	BH	0.00
2018	DP	3.00
2018	FR	2.67
2018	TP	16.00

Note

Average number of days of flowering in plots on south‐facing, north‐facing, and combined slopes at a site. Days and percentage extension in flowering time (calculated as: (Combined‐Longer)/Longer) averaged across all sites for (A) all pollinator resources, as well as (B) after removal of species turnover (limited to only including species present on both aspects of a site each year). (C) Difference in the number of days of flowering extension at each site every year ([All resources] − [Resources without turnover]). Negative values reflect cases where overall community flowering time extension across aspects was lower than the average extension due to within‐species differences.

### 3.3 Q3: diversity components of extension

Species turnover accounted for some of the difference in aspect flowering time. When turnover was removed in the extension calculations, flowering time extension decreased by an average of 5 days (Table 2C). However, there was some variation in the turnover effect, and not all sites exhibited reduced flowering time extension when species turnover was removed (Figure 4c, Table 2C). Additionally, two sites decreased in the extension metric in 2015 with turnover, indicating that intraspecific differences in flowering time between aspects were greater than interspecific differences in these cases. Full tables of site by year extension metrics are included in the supplementary information (Table S1).

**FIGURE 5:**
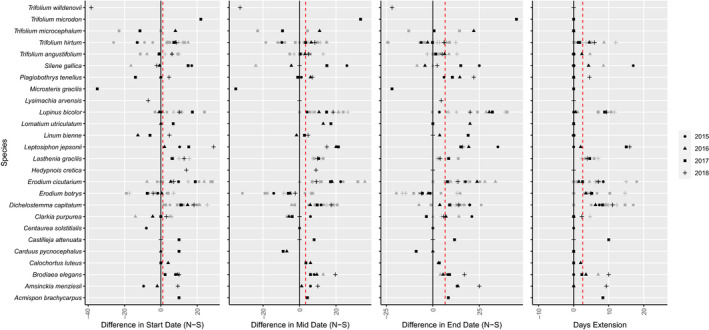
Species differences and flowering extension by site and year. Difference in mean (a) start (5% flowering), (b) mid (50% flowering), and (c) end (95% flowering) dates between north‐ and south‐facing slopes (North date – South date), and (d) Number of days of flowering extension (combined duration ‐ longer slope duration) for pollinator resource species present on both slopes at a site. Gray points represent difference within each site within a given year, and black points are mean difference among sites each year. Symbols indicate years: 2015 (circles), 2016 (triangles), 2017 (squares), and 2018 (crosses). Red dashed lines indicate the mean across species (weighted 1 value (mean across years) per species). In panels A‐C, positive values indicate earlier timing on south‐facing slopes, and later timing on north‐facing slopes. Values from *Lasthenia gracilis* are from 2017 and 2018 experimental plots where genotypic variation was controlled

A total of 32 pollinator resource species were present across both aspects, in one or more plots. These species showed a variety of timing responses to aspect. The mean difference across species was 1.0 days for start dates, 3.3 for midflowering dates, and 6.6 for end dates (Figure 5a–c). These differences resulted in a mean extension of 2.6 days averaged across species (Figures 4 and 5d). Most (75%–81%) of the differences between north and south slope flowering times were positive, revealing later flowering start, mid, and end dates on north‐facing slopes. However, this was not always the case, and some species–year combinations yielded no difference, or an *earlier* timing on north‐facing slopes.

### 3.4 Q4: population plasticity contributions to differences across aspect

Subplots of *Lasthenia gracilis* genotypes also exhibited differences in timing due to aspect (*p* < .001, Table 3; Figure 5 ‐ *L. gracilis* points). This difference reveals population mean plasticity in flowering time in response to abiotic differences between aspects. This flowering difference across aspects extended the flowering time of *Lasthenia* an average of 4.5 days (Table 3; Figure 5d—*L. gracilis* points). Unfortunately, no naturally occurring *L. gracilis* occurred on the south‐facing slopes in the study sites during this experiment, so we could not compare whether the effect of aspect in the experimental plots was different than in the natural communities for *L. gracilis*. However, this pattern of complementarity follows that observed for other species within the main plots. Genotypic differences in phenological phase timing between aspects would increase the extension of flowering time if it led to complementarity in natural communities.

**TABLE 3:** Duration of season and extension metrics split by species

Year	Mean south	Mean north	Mean combined	Mean days extension	Range of days extension	Mean extension
Pollinator resource species						
2015	17.7	25.9	30.9	3.77	1.6–5.8	14.1%
2016	16.2	21.9	28.2	2.37	0.7–3.5	14.1%
2017	16.9	19	26.6	3.59	0.8–6.3	16.0%
2018	21.2	22.5	31.7	5.35	4.5–6.7	19.6%

Note

Average number of days of flowering for species on south‐facing, north‐facing, and combined slopes at a site. Duration is defined as number of days from mean start dates (at least 5% in flower) to mean end dates (at least 95% in flower) within each species. Percentage extension in flowering time is calculated as: ((Combined‐Longer)/Longer) for each species.

## 4 DISCUSSION

Our study establishes that topographic heterogeneity can lengthen flowering time duration on a landscape and may therefore support species interactions in responding to climate changes. Topographic positioning, and the resulting differences in abiotic and biotic conditions, led to complementarity in flowering time of pollinator resources across the landscape. The lengthened duration of resource availability on the landscape reveals the potential of diverse topography to support both pollinator and plant species.

Temperature accounted for approximately 41% of the variation in midflowering dates (25%–52% each year), with flowering dates advancing approximately 3.4 days per 1°C average temperature increase during the March–May flowering season (Figure 3; marginal *R* ^2^ of midflowering dates × temperature: 0.41). This strong negative relationship is consistent with temperature being a driver of flowering time in temperate regions, and matches trends observed in other systems across both space (e.g., Timberlake et al., 2019) and time (e.g., Miller‐Rushing & Primack 2008). The observed sensitivity of the flowering time response to temperatures across space (3.4 days per 1°C) is congruent with previous reports of plant flowering of ~3 days earlier for each 1°C increase in temperatures over time (e.g., Miller‐Rushing & Primack 2008).

The timing of flowering across microsites resulted in complementarity in flowering resources within a site. Our results show an average of 8%–15% total extension (depending on the year), yielding an average of 4–8 additional days of pollinator resource availability within small‐scale landscape features (1,000–2000 m^2^ area). This scale is important as small pollinators may only move very short distances, and the linear distances between aspects in this study (≤100 m) were within foraging range of small insect pollinators (Zurbuchen et al., 2010). The extension of flowering duration was due, in part, to intraspecific differences across aspects (0%–8.6%), with an additional 1%–14% extension each year due to turnover (Figure 4b,c, Table 2). Therefore, both intraspecific differences and species turnover are important determinants of resource duration across the landscape.

These calculations of the flowering time extension are conservative estimates, and the benefits of heterogeneous topography on the landscape may be much greater than reported here. If extension calculations were instead based on the shorter flowering period of the two paired aspects, the mean season extension observed at a site would be 12–22 days (26%–47%) depending on the year. We also defined start and end dates as the date of 5% and 95% flowering, respectively. If absolute start and end dates of flowering were used instead, the extension estimates may be even greater.

Other properties of landscape patches can work synergistically with heterogeneous topography to extend flowering time. The effect of aspect was dependent on site, a pattern that was consistent across years (Site:Aspect *p* < .001 and Year:Site:Aspect interaction NS; Table S2). This indicates that there are properties that make some sites more conducive to phenological complementarity and extension. Both abiotic and biotic differences between aspects in a site may determine the magnitude of flowering time complementarity, and therefore overall flowering duration. Turnover between aspects at a site explained 26% of the variation observed in community flowering time extension (Figure 4b). Once turnover was removed in extension calculations, the extension of flowering time with paired aspects was reduced to zero in 8 out of 16 cases (Figure 4). To further explore this finding, we examined site richness and determined that sites with higher overall richness also exhibited higher flowering resource extension (correlation test, *p* = .02). In addition, as temperature is related to flowering time (Figure 3), sites with larger temperature differences between aspects also resulted in the more flowering time complementarity and extension.

Individual species responses were variable (Figure 5), but in a majority of cases (75%) the presence of a species on both aspects within a site led to an extension of flowering time for that species (Figure 5d, Table 2). This indicates that topography can extend the duration of flowering within a species, which is important for more specialized pollinators. Intraspecific extension across aspects is due in part to different abiotic cues across topography, such as degree‐day accumulation, as warmer plots (e.g., south‐facing aspects) exhibited earlier flowering in most cases (Figure 3). However, in some cases north‐facing slopes exhibited earlier flowering times (Figure 5), indicating that the differences in intraspecific flowering across aspects are a bit more complex. This may be due to differences in plant density (e.g., Schmitt 1983), or in soil properties, moisture, shading, or disturbance (e.g., Heinrich 1976). Likely multiple factors are at play in determining flowering extension across topography, and abiotic and biotic factors may interact to determine extension for each species.

Gaps between flowering on paired aspects did occur for individual species and should be taken into consideration. As differences in conditions between patches on the landscape become more distinct, flowering resource timing will be extended, but only up to the point at which a gap in time between the two flowering curves occurs. High heterogeneity in abiotic conditions with a continuum of temperature and moisture environments should maximize the extension of pollinator resources and minimize flowering gaps, as intermediate condition patches can fill floral resource “valleys” between patch type extremes (e.g., Aldridge et al., 2011).

The timing differences exhibited by the *L. gracilis* experimental plots indicate phenotypic plasticity for flowering time in response to different abiotic conditions across aspects (Table 4). However, species may also exhibit genotypic differentiation at this scale, and genotype by environment interactions may account for some of the larger magnitude of extension exhibited by some species (Figure 5d) and complexity in species responses observed. Reciprocal transplant studies are necessary to determine unequivocally whether genotypic variation contributes to intraspecific differences between sites.

**TABLE 4:** Duration of season and extension with fixed genotypes

Year	Mean south	Mean north	Mean combined	Mean days extension	Mean extension
*Lasthenia gracilis*					
2017	16.2	20.2	25.6	5.4	28.3%
2018	23.7	14.8	27.6	3.9	16.8%

Note

Average number of days of flowering in *Lasthenia gracilis* plots on south‐facing, north‐facing, and combined slopes at a site. Duration is defined as number of days from mean start dates (at least 5% in flower) to mean end dates (at least 95% in flower) for experimental plots of *L. gracilis*. Percentage extension in flowering time was calculated as: (Combined‐Longer)/Longer) for all sites, in both years with *L. gracilis* experimental plots.

The annual timing of life cycle events determines when and how species interact and is important for ecosystem function. Pollinators require readily available resources within an appropriate foraging distance at specific time periods in order to complete their life cycles. Likewise, animal‐pollinated plant species need pollinators to be present at the right time for successful reproduction. Lengthened flowering seasons support pollinator biodiversity (Russo et al., 2013) and can improve plant pollination services (Kremen et al., 2007). Therefore, the presence of topographic heterogeneity may serve to support both plant and pollinator biodiversity in natural systems by extending flowering time on the landscape.

Pollinators rely on both presence and abundance of resources throughout the season (Aldridge et al., 2011) and have varying nutritional needs (Vaudo, Tooker, Grozinger, & Patch, 2015). Our study examines the ability of topoclimate to extend the flowering time of plant communities and species, focusing on the presence of resources across the season, but not the abundance or quality of those resources. The quantity of resources flowering during the season was quite variable, and species and floral abundances differed across aspects. The quality and nutritional content of floral resources can vary due to differences in abiotic conditions and species present (Vaudo et al., 2015). It will therefore be important to consider resource quality, along with abiotic differences and biotic diversity, in determining the potential of topographic gradients to support pollinators across the flowering season.

Our findings reiterate that topographic heterogeneity is important to consider in determining the impacts of climate change. The average temperature difference of 3°C found between north and south slopes is roughly equivalent to the lapse rate for 500 m elevation difference or about 5° latitude in flat landscapes (Barry, 2008; Bennie et al., 2008), and to the amount of warming that may occur over the next 50 years (IPCC 2014). Studies have indicated that topography may create important microrefugia for species as the climate changes (Dobrowski, 2011). Microclimatic effects on plant phenology (e.g., due to topographic positioning) may allow animals to move across the landscape as resources become available, increasing the duration of resources on the landscape as a whole (Hindle et al., 2015). Our study highlights the importance of topographic heterogeneity as a means of extending flowering time on a landscape, and thus potentially supporting species interactions.

Topographic heterogeneity may serve to buffer some impacts of shifts in phenology with climate change, yielding adaptive capacity. A longer flowering phenology across a landscape may aid both pollinator and plant species to cope with these changes by buffering the magnitude of asynchrony at the landscape level (Olliff‐Yang et al., 2020). Recent modeling predicts duration as one of the most important factors in species persistence for plants and pollinators with shifts in phenology (Franco‐Cisterna, Ramos‐Jiliberto, de Espanés, & Vázquez, 2020), and topographic diversity has been predicted to reduce the chance of mismatch for some species (Hindle et al., 2015). Conserving, restoring, and maintaining high species diversity across topography may therefore support species interactions by buffering the impacts of mutualism asynchronies with climate change.

## CONFLICT OF INTEREST

None declared.

## AUTHOR CONTRIBUTIONS

**Rachael L. Olliff‐Yang:** Conceptualization (lead); data curation (lead); formal analysis (lead); funding acquisition (lead); investigation (lead); methodology (lead); validation (lead); visualization (equal); writing–original draft (lead); writing–review and editing (equal). **David Ackerly:** Conceptualization (supporting); formal analysis (supporting); funding acquisition (supporting); methodology (supporting); visualization (equal); writing–original draft (supporting); writing–review and editing (equal).

### Open Research Badges

This article has earned an Open Data Badge for making publicly available the digitally‐shareable data necessary to reproduce the reported results. The data is available at https://doi.org/10.6078/D1KX30; https://github.com/rlolliff/Flowering-time-across-topography.

## Supporting information

Figure S1

Figure S2

Table S1

Table S2

## Data Availability

Data are archived and publicly available on Dryad, https://doi.org/10.6078/D1KX30; Data analysis code R scripts are available at: https://github.com/rlolliff/Flowering-time-across-topography
