# Supplementary material for: Topographic heterogeneity lengthens the duration of pollinator resources
Source: Ecol Evol. 2020 Aug 10;10(17):9301–12. doi: 10.1002/ece3.6617 (PMC7487246; doi:10.1002/ece3.6617)
Supplement: Supplementary file 1 — Figure S1 [file ECE3-10-9301-s001.docx]

**Figure S1.** Growing degree day accumulation differences between south (light red) and north (dark blue) facing aspects in 2016. Faster increase (steeper slopes) reveal warmer slopes. Panel titles indicate site: Bechtel House, Double ponds, Hill 1521, and Turtle ponds. DOY 100 = 9 April, DOY 200 = 18 July, DOY 300 = 26 Oct. Degree days calculated with base temp of 5˚C; as ((Max T + Min T)/2) – 5)). Data presented is from 2016, as temperature data was only recorded from March - June in other years. Across all years south aspects accumulated more growing degree days during the growing season than N aspects more often than expected by chance (binomial test, p <0.001).
