# Supplementary material for: Topographic heterogeneity lengthens the duration of pollinator resources
Source: Ecol Evol. 2020 Aug 10;10(17):9301–12. doi: 10.1002/ece3.6617 (PMC7487246; doi:10.1002/ece3.6617)
Supplement: Supplementary file 2 — Figure S2 [file ECE3-10-9301-s002.docx]

**Figure S2** - Temperature and rainfall accumulation in (A) 2015, (B) 2016, (C) 2017, and (D) 2018. Day of water year indicates days since 1 October of the previous year (e.g. 1 Oct 2015 begins the 2016 water year), as defining the water year in California (mediterranean- type climate with wet season beginning in October). Black line is rainfall accumulation in mm, and grey line is temperature accumulation in growing degree days (thermal accumulation above base temperature of 5 ˚C). Steeper slopes indicate periods of faster accumulation. Dashed connecting lines show periods of missing data. Missing rainfall data in 2018 water year was extrapolated based on total rainfall for the year. Vertical dotted line indicates DOY = 1 (January 1^st^) of each year, for reference to other figures.
